# *Haemophilus aphrophilus* Endocarditis after Tongue Piercing

**DOI:** 10.3201/eid0808.010458

**Published:** 2002-08

**Authors:** Hossein Akhondi, Ali R. Rahimi

**Affiliations:** * Mercer School of Medicine, Savannah, Georgia, USA

**Keywords:** piercing complications, *Haemophilus aphrophilus*, endocarditis

## Abstract

Piercing invades subcutaneous areas and has a high potential for infectious complications. The number of case reports of endocarditis associated with piercing is increasing. We studied a 25-year-old man with a pierced tongue, who arrived at Memorial Health University Medical Center with fever, chills, rigors, and shortness of breath of 6 days duration and had an aortic valvuloplasty for correction of congenital aortic stenosis.

Body piercing poses a risk for serious disease. Because it invades subcutaneous areas, piercing has a high potential for infectious complications. Such complications result from introduction of skin or mucous membrane microflora into subcutaneous tissue or from the ongoing presence of colonies of these microflora at the piercing site. Pain, edema, and prolonged bleeding may occur immediately after piercing (1), and a cyst, scar, or keloid may form at the piercing site. In various surveys, the rate of earlobe piercing infections alone has been estimated at 11% to 24%. Skin lesions or anatomic abnormalities at the site of piercing, as well as valvular heart disease, are risk factors for complications (2). Staphylococcal endocarditis of the mitral valve after nasal piercing (3), *Neisseria* endocarditis after tongue piercing (4), and *Staphylococcus epidermidis* endocarditis and mastitis following nipple piercing have been reported (5). Even though a consistent correlation is not known between piercing and endocarditis, the number of case reports is increasing, and a correlation may well exist.

Persons at high risk for complications should be treated with preventive antibiotics, just as persons at high risk for complications receive antibiotic treatment before dental procedures. The correlation between dental procedures and endocarditis has been reviewed by Van der Meer et al., who prospectively examined all cases of infective endocarditis in the Netherlands over a 2-year period (6). Of 427 patients who had been hospitalized, 64 had previous dental or other procedures in the preceding 3 months. Using 48 of these 438 patients as study cases (only 48 patients met the qualification of having native-valve and cardiovascular anomalies that increased their risk of getting endocarditis, these researches found no significant difference in presence of dental procedures between patients and matched controls without endocarditis (odds ratio 1.2, 95% confidence interval 0.03 to 2.3). Two other studies (7,8) reported similar results. No study has examined the correlation between piercing and endocarditis.

In the United States, body piercing, which is becoming increasingly common, is mainly performed by unlicenced practitioners. Only 26% of states have regulatory authority over tattooing establishments, and only six of these states exercise authority over body-piercing establishments. Piercing occurs in regulated and unregulated shops, department stores, jewelry shops, homes, or physicians’ offices. Generally no antibiotic is used, and sterilization methods vary. Studies show that ear piercing can cause cephalic tetanus (a local form of tetanus caused by wounds or other head and neck infections) (8), *Pseudomonas* infections, or perichondrial auricular abscesses, especially with *Pseudomonas aeruginosa.* Tongue or oral piercing can cause Ludwig’s angina (2,9,10) or may be complicated by normal oral flora, such as *Haemophilus aphrophilus*, as in this case. Genital piercing may result in *Escherichia coli* infection and may increase the risk for sexually transmitted diseases through tissue damage and exposure and unwanted pregnancy because of condom rupture (11). Systemic infections, such as toxic shock syndrome or sepsis, have also been reported (10). Among noninfectious cases, granulomatous perichondritis of the nasal ala, sarcoidlike foreign body reaction from multiple piercing, paraphimosis from a distal penis pierce, and speech impairment, together with difficulty in chewing and swallowing from oral jewelry, have been reported (1,2,9,10). Metal-associated problems include allergy (especially to nickel), eczematous rash, and lymphocytoma (2,9,10,12). We describe an incidence of *H. aphrophilus* endocarditis following tongue piercing.

## Case Report

A 25-year-old man arrived at Memorial Health University Medical Center with fever, chills, rigors, and shortness of breath of 6 days duration. He had a history of aortic valvuloplasty at 8 years of age for correction of congenital aortic stenosis. At admission, the patient had fever of 38.9°C and a grade III/VI ejection systolic murmur accompanied by a grade II/VI diastolic blowing murmur best heard in the left sternal border area. The oral cavity was pink, and no inflammation or exudates were noticed on the pharynx. The middle portion of the tongue had been pierced, and a bispherical stud was in place (Figure). The piercing was performed 2 months before onset of illness. Extensive tattoos on the shoulders, arms, and upper torso dated back 3 years. The patient had previous dental work done but always with antibiotic prophylaxis.

**Figure:**
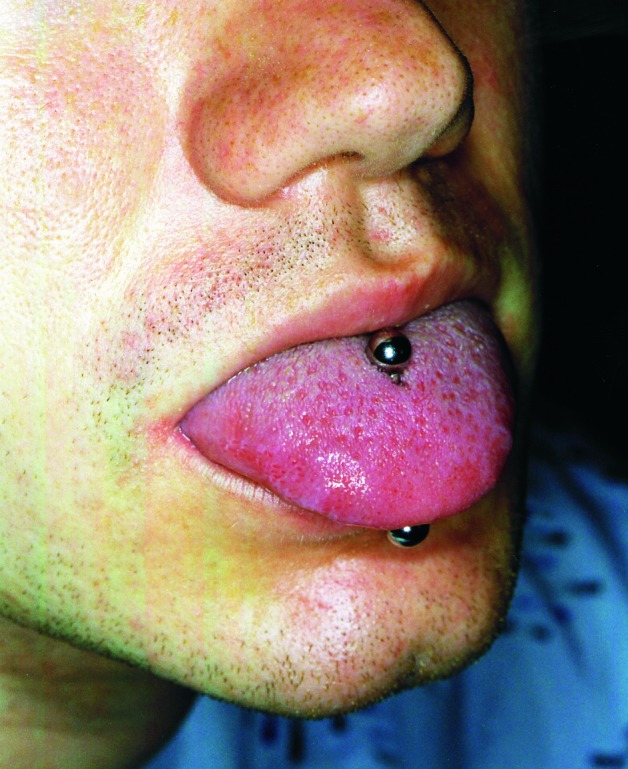
The tongue pierce of the man from the case study. The stud was bispherical metal inserted without anesthesia or preparation. Although the stud was removable, the patient had not removed it. The area around insertion was clean with no local sign of infection when the stud was removed; the tongue was not inflamed or painful.

Laboratory tests showed erythrocyte sedimentation rate of 41 mm/hr (normal rate, 0–15 mm/hr) and elevated C-reactive protein of 5.1 mg/dL (normal level 0–1). Transthoracic echocardiography was not conclusive; a transesophageal echocardiogram showed remnants of a bicuspid and deformed aortic valve with multiple vegetative lesions. Blood cultures were obtained, and the patient was started on triple antibiotics (ampicillin, nafcillin, and gentamycin). Wet preparation and acridine orange stain of the blood specimen showed gram-negative pleomorphic rods. Two of the conventional chocolate-agar cultures turned positive approximately 4 days after incubation and were consistent with *H. aphrophilus* (*β*-lactamase negative, lactose fermenting, and Mannose fermenting). The stud culture was also positive for *H. aphrophilus*. Antibiotics were modified because of sensitivity to ceftriaxone and gentamycin, and the patient was discharged to complete the 6-week course through a peripherally inserted central catheter line at home. Aortic valve replacement was recommended after completion of antibiotic therapy, but the patient did not return for treatment.

## Conclusions

Our case demonstrates *H. aphrophilus* endocarditis possibly caused by tongue piercing (or as a complication of the ongoing presence of the stud) in a patient with congenital heart disease. Colonization around the stud likely caused bacteremia and endocarditis. *H. aphrophilus* is commonly isolated from the upper respiratory tracts of humans and animals; however, its prevalence is unknown. In a previous study of piercing complications in patients with congenital heart disease (13), 43% of the study population had earlobe piercing; of these, 6% took antibiotics before piercing. Twenty-three percent of patients had piercing-related infections 1 week to 3 years after piercing. Most infections were local skin infections; no endocarditis was reported in that study.

Until prospective randomized studies shed light on the relationship between piercing and endocarditis, prophylactic measures are indicated and should be formulated, particularly for persons at high risk, e.g., those with structural heart diseases.
